# The Role of Aromatic Amino Acids in Polycystic Ovary Syndrome through Patients’ Blood Metabolic Profiling: A Systematic Review of the Past Five Years

**DOI:** 10.1021/acs.jproteome.4c00937

**Published:** 2025-04-17

**Authors:** Apostolos Gkantzos, Stavros Kalogiannis, Olga Deda

**Affiliations:** † Department of Nutritional Sciences and Dietetics, International Hellenic University, 57400 Thessaloniki, Greece; ‡ Laboratory of Forensic Medicine & Toxicology, Department of Medicine, Aristotle University of Thessaloniki, 54124 Thessaloniki, Greece

**Keywords:** Polycystic Ovary Syndrome, Aromatic Amino Acids, Phenylalanine, Tyrosine, Tryptophan, Insulin Resistance, Inflammation, Metabolomics

## Abstract

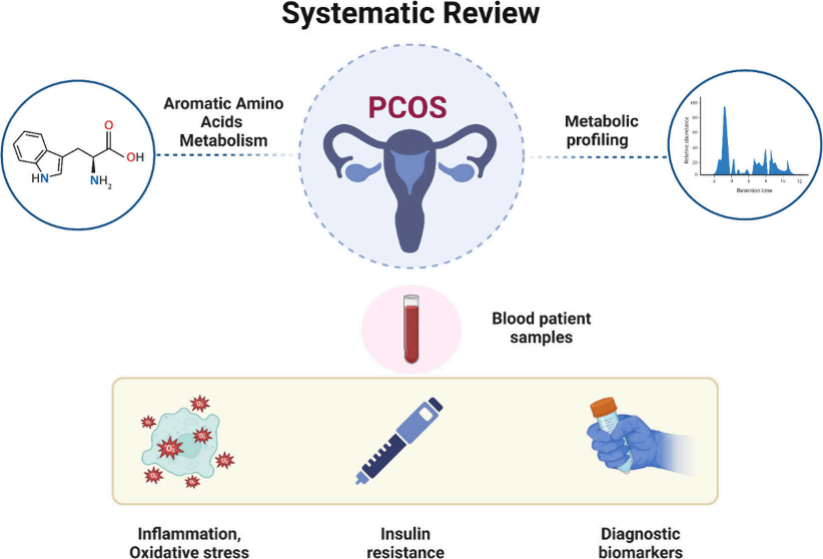

Polycystic ovary syndrome (PCOS) is a common endocrine and metabolic disorder in women of reproductive age that encompasses a multitude of signs and symptoms, including hyperandrogenism, polycystic ovarian morphology, ovulatory dysfunction, and insulin resistance. The study aims to explore the role of aromatic amino acid (AAA) disorders in the syndrome. A systematic search on the databases Scopus, PubMed, and Google Scholar until 20 July 2024 over the past 5 years regarding metabolomic studies on PCOS patients’ blood and the status of AAAs resulted in 12 related papers. Our review showed that AAA metabolic pathways are dysregulated, and their levels in the blood serum and plasma of PCOS patients in most studies are elevated due to inflammation and oxidative stress which, assisted by gut dysbiosis, give rise to insulin resistance that develops into PCOS. AAA abnormalities can also directly induce the defining symptoms of the syndrome through diminished neurotransmitter availability and impaired signaling. According to our review, AAA perturbations are detected in every stage of PCOS pathophysiology, making them valuable biomarkers for early diagnosis and management of the syndrome. Further investigation of the biological function, role, and impact of AAAs, probably alongside other metabolites, including BCAAs, could lead to the discovery of new tools for preventing and managing PCOS symptoms.

## 1 Introduction

Polycystic ovary syndrome (PCOS) is an endocrine and metabolic disorder affecting women of reproductive age and its global prevalence is estimated to range from 6% to 21%, making it the most common endocrine disorder for women of this age group.^1^ As the term “syndrome” implies, PCOS is a disorder characterized by many signs and symptoms without a clear unifying cause, making universal treatment elusive, and even consensus on a specific definition within the scientific community is undecided.^2^ In modern clinical practice, there are mainly 2 types of criteria used to diagnose PCOS: the Rotterdam Criteria established in 2003 state that a diagnosis of PCOS is valid when at least 2 of hyperandrogenism, ovulatory dysfunction, and polycystic ovarian morphology coexist, while the criteria proposed by the Androgen Excess and PCOS Society in 2006 suggest that the presence of hyperandrogenism is mandatory and accompanied by either ovulatory dysfunction or polycystic ovarian morphology or both.^2^

The heterogeneity of PCOS is made apparent by the multitude of signs and symptoms characterizing its clinical presentation, some of which are hirsutism, acne, alopecia, insulin resistance, metabolic dysfunction, and mood disorders.^2^ Insulin resistance in particular is estimated to appear in 65–95% of PCOS cases regardless of weight status.^1^ Although there has been some discourse on whether the increased amount of androgens is the source of most woes associated with the syndrome, recent research findings indicate that insulin resistance could be the proverbial egg coming before the chicken of hormonal imbalances and corresponding metabolic and ovulatory disorders.^3−5^ Insulin resistance is a complex pathological state leading to hyperinsulinemia and impaired glucose transportation in target tissues, also correlated with conditions such as metabolic syndrome, cardiovascular diseases, and type 2 diabetes mellitus.^6^ A wide array of factors contribute to its establishment and perpetuation. A common denominator is nutrient oversupply and the derived mechanisms and responses surrounding it such as oxidative stress and inflammation,^7^ both of which are implicated in PCOS pathophysiology.^8^

Aromatic amino acids (AAAs) phenylalanine, tyrosine, and tryptophan are called essential because they cannot be naturally synthesized in the human body but are only obtainable through diet. Phenylalanine is the precursor of tyrosine which in turn is a precursor to neurotransmitters dopamine, epinephrine, and norepinephrine while tryptophan is metabolized to serotonin and also takes part in the tryptophan–kynurenine pathway.^9^ Increased concentrations of these 3 amino acids and dysregulations in their metabolic pathways have been linked with complications such as insulin resistance, obesity, inflammation, and mood disorders.^10,11^ A study by Würtz et al. proposed that phenylalanine and tyrosine in men as well as phenylalanine in women could predict the onset of insulin resistance as far as 6 years in the future suggesting that aromatic amino acid impairments predate its development.^12^ A 7.5-year longitudinal study in prepubescent girls showed that hyperinsulinemia develops during puberty before normalizing again in early adulthood and phenylalanine along with tyrosine were positively associated with the condition during all stages.^13^ In another prospective study undertaken in a Japanese population phenylalanine and tyrosine were higher in overweight and obese adults and at the same time tyrosine was positively associated with insulin resistance in that group but not in underweight and normal-weight adults.^14^ Moreover, the tryptophan–kynurenine metabolic pathway has been correlated to obesity-induced inflammation in adults^15^ while also playing an important role in gut microbiota-related health issues.^16^ The value of AAAs as biomarkers has also been demonstrated in a study conducted on a Korean population in which they showed potential in predicting metabolic diseases and hypertension in adults.^17^ Furthermore, catecholamines and serotonin, the products of AAA metabolism, are neurotransmitters which are implicated in mood disorders and reproductive health, two significant aspects of PCOS pathology.^18^ All of these aromatic amino acid-related pathologies interact with each other and, with insulin resistance at the forefront, plague PCOS patients leading to a poor quality of life for the affected women.^19^

Metabolomics is the comprehensive analysis of metabolites in a biological specimen.^20,21^ It is a rapidly growing field making use of techniques such as nuclear magnetic resonance spectroscopy (NMR) and liquid or gas chromatography in tandem with mass spectrometry (LC-MS/GC-MS) to detect metabolites and characterize entire metabolic imprints in target tissues.^22,23^ A key strength of metabolomics lies in the fact that it is a fundamentally phenotype-driven approach with high sensitivity allowing researchers to get real-time personalized information from the target matrix.^24^ Additionally, liquid chromatography–mass spectrometry -based methods have claimed a spot as methods of choice for the diagnosis of biochemical hyperandrogenism in PCOS according to the 2023 International Evidence-based PCOS Guideline.^25^ A wide array of samples can be used for metabolomics analysis, some of the more popular ones being plasma, serum, and urine.^26^ The nature of PCOS as a disorder affecting the reproductive system has made follicular fluid a common target for metabolomics analyses.^27−29^ However, blood samples are easily collected fluids that provide generalized information about the metabolic state of the organism at the time of collection without having to be subjected to extensive catabolic processes.^26^ Metabolomics analyses conducted in blood and other matrices have already hinted at the potential significance of AAAs in the pathophysiology of PCOS.^27,28,30^

Taking into account all of the above, we hypothesized that aromatic amino acids could be metabolites of interest in polycystic ovary syndrome because of their association with key metabolic and hormonal disturbances of the syndrome. The aim of the present systematic review is to record the recent data of the past 5 years regarding the role of aromatic amino acids in the pathophysiology of polycystic ovary syndrome through the use of metabolomics in human blood samples.

## 2 Materials and Methods

The current systematic review was conducted based on the procedure recommended by the Preferred Reporting Items for Systematic Reviews and Meta-Analyses (PRISMA) guidelines. PROSPERO registration was not applicable for metabolomics-based studies [https://www.crd.york.ac.uk/prospero/#aboutpage]. A preliminary search was performed on the databases Google Scholar, Pubmed, and Scopus from July 1, 2024 to July 14, 2024 to explore potential areas related to polycystic ovary syndrome and metabolomics, that could serve as focal points of the current review. The exact search queries for each database were as follows:

Scopus: (TITLE (“polycystic ovary syndrome”) AND TITLE-ABS-KEY (metabol* OR “metabolic profil*” OR metabonomics) AND ALL (serum OR plasma) AND ALL (human OR patients)) AND PUBYEAR > 2017 AND PUBYEAR < 2025 AND (EXCLUDE (DOCTYPE, “re”)) AND (LIMIT-TO (LANGUAGE, “English”)).

Pubmed: (((polycystic ovary syndrome [Title]) AND (metabolo*[Title/Abstract] OR metabolic profil*[Title/Abstract] OR metabonomics[Title/Abstract]) AND (serum OR plasma) AND (human OR patients).

Google Scholar: intitle:(“polycystic ovary syndrome”) AND [tiAb]: (metabolo* OR “metabolic profil*” OR metabonomics) AND (serum OR plasma) AND (human OR patients) -review.

Regarding the Google Scholar search, the setting to search for results only in English was also checked. For both the Google Scholar and PubMed searches, the year range was set from 2018 to 2024 and additional filters for PubMed were to show results that were only clinical trials or randomized control trials. Using these search queries and filters, the number of results yielded by Scopus, PubMed, and Google Scholar were 170, 17, and 69 respectively, which brought the total number to 256 results. Inclusion criteria were studies conducted on human patients with polycystic ovary syndrome using metabolomic techniques. After eliminating double entries, reviews, nonmetabolomic studies, and studies that were not conducted on humans, we ended up with 36 articles that researched polycystic ovary syndrome using metabolomic techniques. Upon comprehensively examining the abstract of each of these articles, we decided that the focus of the current systematic review shall be the role of aromatic amino acids on polycystic ovary syndrome through patients’ blood metabolic profiling. A second search was conducted in the databases Google Scholar, PubMed, and Scopus with terms relating to the current review’s subject, from July 17, 2024 to July 20, 2024. The exact search queries for each of the databases were as follows:

Scopus: (TITLE (“polycystic ovary syndrome”) AND ALL (“aromatic amino acid*” OR tryptophan OR tyrosine OR phenylalanine) AND ALL (metabolo* OR “metabolic profil*” OR metabonomics) AND ALL (human OR patients) AND ALL (blood OR serum)) AND PUBYEAR > 2017 AND PUBYEAR < 2025.

PubMed: (((((“polycystic ovary syndrome”[Title]) AND (“aromatic amino acid*” or tryptophan or tyrosine or phenylalanine)) AND (metabolo* OR “metabolic profil*” OR metabonomics)) AND (human OR patients)) AND (blood OR serum)).

Google Scholar: intitle:(“polycystic ovary syndrome”) AND (aromatic amino acid* OR tryptophan OR tyrosine OR phenylalanine) AND (metabolo* OR metabolic profil* OR metabonomics) AND (human OR patients) AND (serum or blood).

**Figure 1:**
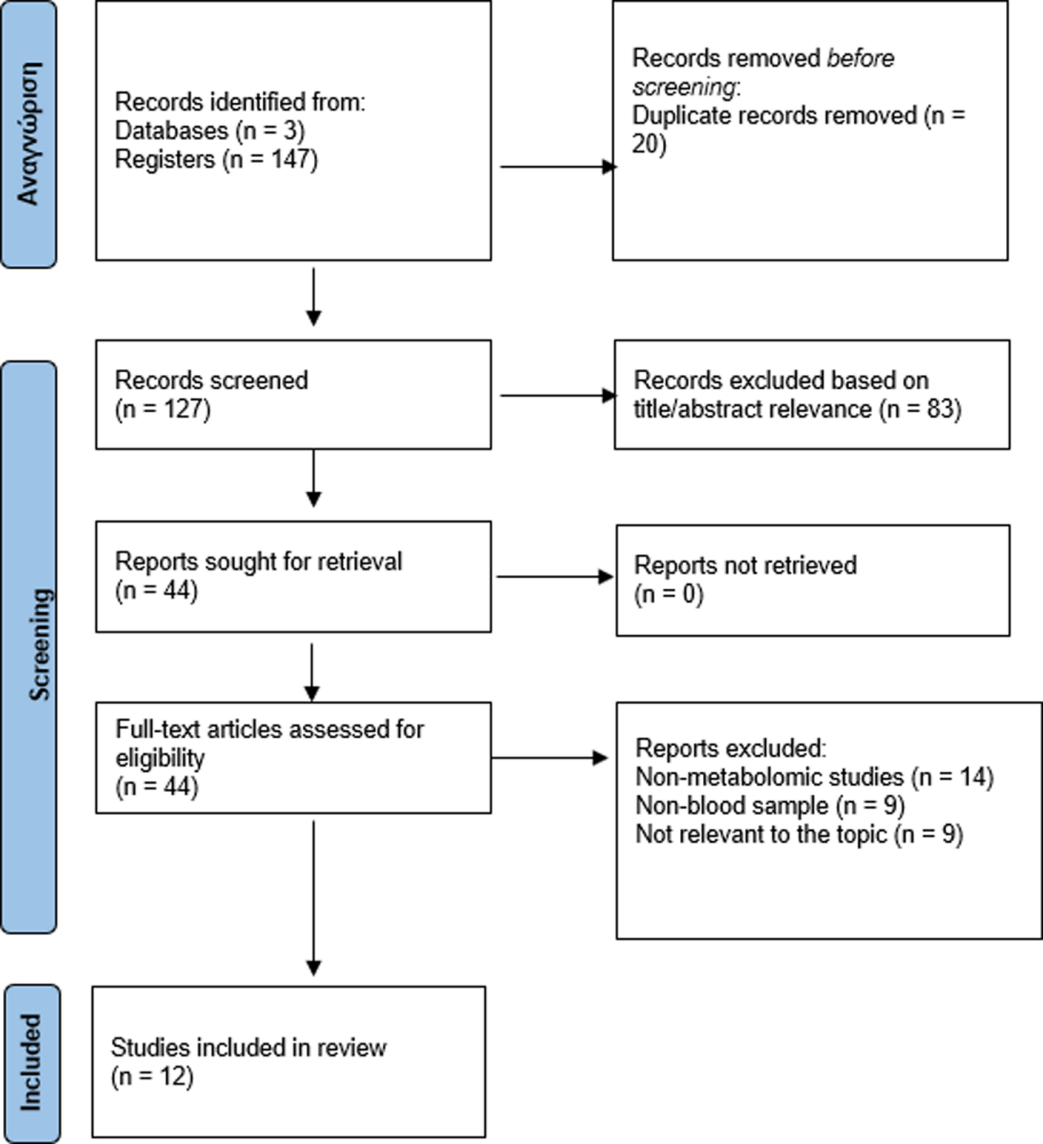
PRISMA flowchart detailing the search process.

Regarding the Google Scholar search, the setting to only search for results in English was ticked on, and for both the Google Scholar and the PubMed searches, the year range was set from 2018 to 2024. Using these search queries, the number of results yielded by the Scopus, PubMed, and Google Scholar databases were 93, 5, and 49 respectively, which brought the total number to 147 results. Following a comprehensive examination of the title and abstract in each of these articles and eliminating double entries, nonhuman studies, reviews, and book chapters, we ended up with 44 articles. The three of us then assessed the full text of each of these 44 articles to ensure that they qualified for use in the present systematic review. The inclusion criteria were studies using 1) metabolomic techniques, 2) blood samples, 3) patients with polycystic ovary syndrome, and 4) whose results highlighted a potential role for aromatic amino acids in the disease. The current systematic review was not registered with PROSPERO as its primary focus is on metabolomics data. We assessed the study quality using the National Institutes of Health (NIH) Quality Assessment Tool for Observational Cohort and Cross-Sectional Studies. To guarantee methodological robustness and minimize bias, we included only studies rated as ‘Good’ or ‘Fair’. We also considered statistical adjustments for potential confounders in interpreting the findings. Figure 1 shows a flowchart detailing the process of the second search following the PRISMA guidelines:

## 3 Results

**Figure 2:**
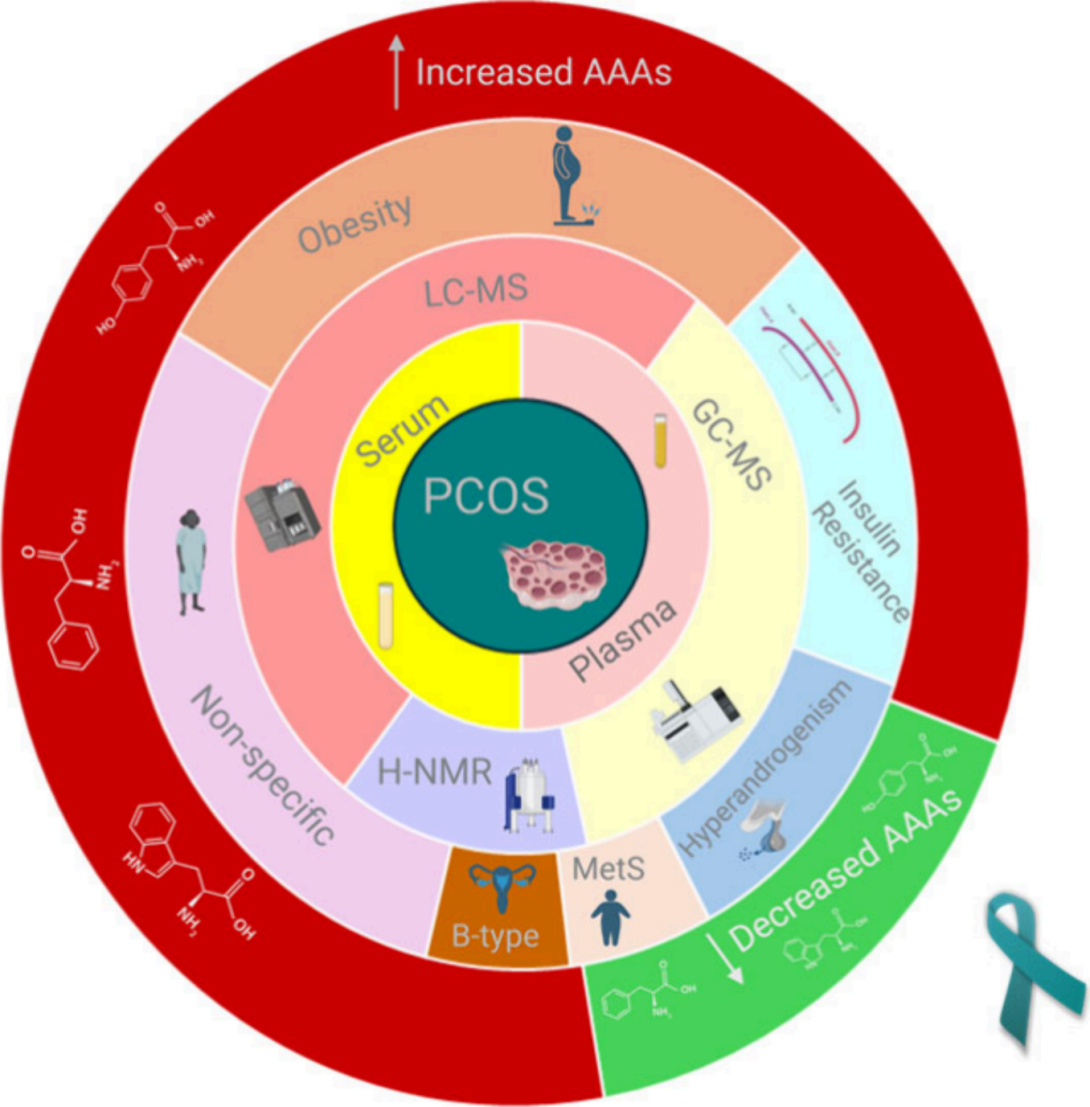
Graphical summary of the main characteristics of the bibliographic search on modified aromatic amino acid levels in the blood of polycystic ovary syndrome (PCOS) sufferers. A systematic review on PCOS was conducted. The studied samples were serum and plasma analyzed by liquid chromatography–mass spectrometry (LC-MS), gas chromatography–mass spectrometry (GC-MS), and proton-nuclear magnetic resonance spectroscopy (^1^H NMR). Prominent phenotypes were obesity, insulin resistance, hyperandrogenism, metabolic syndrome, and B-type PCOS although in some studies there was not a specific phenotype examined. The vast majority of studies reported increased levels of aromatic amino acids in PCOS patients.

As shown in Table 1, our systematic review included 12 research articles published since 2018 that used metabolomic techniques on human blood samples and highlighted the role of aromatic amino acids (AAAs) in the pathophysiology of polycystic ovary syndrome (PCOS) (Figure 2). Of these articles, 8 were published between 2022 and 2024 and the overwhelming majority were clinical studies with only one being a cross-sectional study. Regarding the number of participants, a total of 1098 PCOS patients were included with the highest number being 223 as reported in the study of Zhang and Hong et al.^37^ and the lowest number being 17 as reported in the 2 articles of Escobar-Morreale and Martinez-Garcia et al.^34,35^

**Table 1:** Reference Table of the Studies Included in the Systematic Review

**Author (Date)**	**Study Design**	**Participants**	**Study aim**	**Sample**	**Metabolomics-based technique**	**Clinical Focus of Studies**	**AAAs status in patients**
Saleh (2023)^31^	Clinical Study	65 PCOS cases	Plasma amino acid (AA) measurement	Plasma	Reverse phase- High-performance liquid chromatography (RP-HPLC)	Nonspecific	Increased AAAs
		65 controls					Enriched Phe metabolism and AAA biosynthesis pathways
Paczkowska et al. (2023)^32^	Clinical Study	208 PCOS cases	Plasma amino acid measurement	Plasma	Gas Chromatography–Mass Spectrometry (GC-MS)	Metabolically healthy obese vs metabolically unhealthy obese	Increased AAAs in PCOS and in the Insulin Resistance and Obesity subtypes
		118 controls	Assessment of AAs in metabolic disturbances			Insulin resistance (IR)	
						Obesity	
						Hyperandrogenism (HA)	
Ye et al. (2022)^33^	Clinical Study	190 PCOS cases	Plasma AA profiling	Plasma	High-Performance Liquid Chromatography–Mass Spectrometry (HPLC-MS)	Obesity	Increased AAAs
		190 controls	Exploration of AAs as biomarkers in different metabolic disorders			Insulin resistance	Enriched Phe metabolism and AAA biosynthesis pathways
						Metabolic syndrome	Tyr consistently elevated across metabolic subtypes
							Phe and Tyr as diagnostic markers in combination with other AAs
Escobar-Morreale and Martinez-Garcia et al. (2023)^34^	Clinical Study	17 PCOS cases	Metabolomics profiling for evidence of sexual dimorphism in fasting metabolomic profiles	Plasma	Proton -Nuclear Magnetic Resonance Spectroscopy (^1^H- NMR)	Obesity (in the context of androgen excess)	Increased Phe
		17 controls	Determination of whether androgen excess in women masculinizes intermediate metabolism				Increased Tyr (vs controls and in obese PCOS vs nonobese PCOS)
		19 healthy men					
Escobar-Morreale and Martinez-Garcia et al. (2023)^35^	Clinical Study	17 PCOS cases	Metabolic profiling for evidence of masculinization of postprandial metabolism in PCOS	Serum	^1^H- NMR	Obesity (in the context of androgen excess)	Increased Phe and Tryptophan (Trp) in obese PCOS compared to nonobese PCOS
		17 controls					
		19 healthy men					
Yu et al. (2021)^36^	Clinical Study	31 PCOS cases	Identification of metabolic markers in PCOS	Serum	Ultra-Performance Liquid Chromatography–High-Resolution Mass Spectrometry (UPLC-HRMS)	Nonspecific	Enriched Phe metabolism
		31 controls					AAA biosynthesis pathways
Zhang and Hong et al. (2020)^37^	Clinical Study	223 PCOS cases	Metabolic profiling	Serum	GC-MS	Hyperandrogenism	Increased Phe in PCOS vs controls, IR vs controls, and HA vs IR
		64 controls	Diagnostic markers for HA and IR			Insulin Resistance with and without obesity	
Buszewska-Forajta et al. (2019)^38^	Clinical Study	30 PCOS cases	Metabolomic profiling for the determination of a metabolic diagnostic marker	Serum	LC-MS	Nonspecific (Only hyperandrogenic patients)	Increased AAAs
		30 controls			GC-MS		
Rajska et al. (2023)^39^	Clinical Study	35 PCOS cases	Validation of metabolites found in previous study	Serum	LC-MS	Nonspecific (only hyperandrogenic patients)	Decreased Trp from LC-MS
		35 controls			GC-MS		Decreased Phe from LC-MS
							Increased Phe from GC-MS
Wang and Mu et al. (2022)^40^	Cross-sectional study	200 PCOS cases	Investigation of tryptophan–kynurenine pathway in PCOS	Plasma	LC-MS	Nonspecific	Increased Trp levels in normal-weight and obese patients
		200 controls	Detection of metabolic biomarkers				Trp as a biomarker for diagnosis
Zhao and Xiao et al. (2024)^41^	Clinical Study	50 PCOS cases	Changes in metabolic fingerprints of PCOS before and after compound oral contraceptive (COC) treatment	Serum	Ultra-High Performance Liquid Chromatography–High-Resolution Mass Spectrometry (UHPLC-HRMS)	B-Type PCOS with normal body mass index (BMI)	AAA biosynthesis pathways enriched in the treatment group vs controls
		50 controls					
Tang et al. (2019)^42^	Clinical Study	32 PCOS cases	Plasma metabolomic profiling before and after exenatide administration	Plasma	GC-MS	Overweight or Obesity	Increased Phe + Tyr in PCOS
		35 controls					Decreased AAAs after treatment

There was some variety observed in the aim of each study. Saleh^31^ and Paczkowska et al.^32^ aimed to measure the plasma amino acid levels in their participants while Ye et al. measured plasma amino acid levels but also explored amino acids as biomarkers in the different metabolic subtypes of PCOS.^33^ The value of AAAs, as part of an untargeted metabolic profiling, in the diagnosis of PCOS was a topic of contention in quite a few studies^36−38^ as well as in Rajska et al.’s study^39^ who sought to validate their previous research.^38^ Wang and Mu et al. investigated the tryptophan–kynurenine pathway in PCOS alongside the detection of metabolic markers in the said pathway^40^ while Escobar-Morreales and Martinez-Garcia et al. tested their hypothesis of whether androgen excess induces a masculinization effect in different stages of metabolism in women with PCOS.^34,35^ Lastly, 2 studies introduced a treatment stage in their methodology in order to identify the metabolic profile of PCOS patients pre and post-treatment.^41,42^

As mentioned above, the sample used in all of the research papers was blood, and specifically half of them used serum and the other half used plasma. The sample analysis was done using mainly 3 analytical techniques, of which liquid chromatography–mass spectrometry (LC-MS) was used most often at 7 times followed by gas chromatography–mass spectrometry (GC-MS) at 5 times and proton -nuclear magnetic resonance spectroscopy (^1^H-NMR) twice.

Although some of them did not examine a specific clinical subtype, obesity was a common area of focus researched in 5 studies^32−35,42^ and secondarily in Zhang and Hong et al.’s paper.^37^ Another distinct clinical entity was women with insulin resistance and their metabolic profile which were explored in 3 articles^32,33,37^ as well as the metabolic imprint of hyperandrogenism in 2 articles.^32,37^ Escobar-Morreale and Martinez-Garcia,^34,35^ Buszewska-Forajta et al.^38^ and Rajska et al.^39^ included only hyperandrogenic phenotypes in their studies. Other subtypes were metabolic syndrome^33^ and B-type PCOS with normal BMI.^41^

Last but not least, when it came to the aromatic amino acid status in the examined patients, 10 out of 12 studies reported increases in aromatic amino acid levels. Insulin resistance was a common finding in PCOS women across the studies and 3 of them conducted a separate metabolic profiling of the condition.^32,33,37^ Insulin-resistant women with PCOS had increased AAAs compared to insulin-resistant controls.^32^ Of note was the fact that AAAs were significantly increased in insulin-resistant PCOS compared to non-insulin-resistant PCOS.^32,33^ Although generally all 3 amino acids were increased in insulin resistance, tyrosine^32,33^ and phenylalanine^37^ showed the strongest correlations. Interestingly enough, Zhang and Hong et al. categorized their subjects as hyperandrogenic without insulin resistance and nonhyperandrogenic with insulin resistance and reported that phenylalanine was significantly increased in hyperandrogenism compared to insulin resistance.^37^ At the same time in Escobar-Morreale and Martinez-Garcia et al.’s studies obesity was strongly correlated with the increased AAAs, in a metabolic profile that was similar to men.^34,35^

Another important finding was the value of AAAs as diagnostic markers in PCOS. Ye et al. showed that phenylalanine and tyrosine can be part of an amino acid signature that distinguishes PCOS.^33^ Additionally, Wang and Mu et al. noted that metabolites of the tryptophan–kynurenine pathway, tryptophan included, could be compared to hormonal markers when it came to diagnosing PCOS.^40^ Finally, AAAs showed promise as targets for the therapeutic efficacy of exenatide in obese and overweight patients potentially opening up a new road for the future of PCOS treatment.^42^ However, Zhao and Xiao et al. reported the compound oral contraceptives (COCs) enhanced the biosynthetic pathways of AAAs.^41^

## 4 Discussion

In our review, 2 out of the 12 studies employed metabolic pathway and Kyoto Encyclopedia of Genes and Genomes (KEGG) enrichment analyses to investigate the metabolic pathways with a significant role in PCOS.^33,36^ Both studies agreed that the phenylalanine metabolism and aromatic amino acid biosynthesis pathways were enriched in women with PCOS, indicating their importance in the development and perpetuation of the syndrome.

Phenylalanine catabolism involves its conversion to tyrosine by phenylalanine 4-hydroxylase (PaH) and its cofactor 5,6,7,8-tetrahydrobiopterin (BH4),^9,43^ which then may lead to the synthesis of the neurotransmitters dopamine, epinephrine, and norepinephrine^9^ by the action of tyrosine 3-hydroxylase (TyrOH). Tryptophan may be converted to the neurotransmitter serotonin^43^ by the action of tryptophan 5-hydroxylase (TPH) also employing BH4 as a cofactor but more than 95% of its catabolism^40^ takes place in the tryptophan–kynurenine pathway which starts with the oxidation of tryptophan by one of two dioxygenases, namely indolamine-2,3-dioxygenase (IDO) or tryptophan-2,3-dioxygenase (TDO).^9,43^ The production of BH4 and the activation of IDO are initiated by pro-inflammatory signals whose overstimulation has been linked to inflammation-induced complications such as obesity and mood disorders.^11,43,44^ These signals begin with interferon-γ (IFN-γ) which activates the enzymes GTP-cyclohydrolase 1 (GCH1), inducible nitric oxide synthetase (iNOS) and IDO-1.^43^ GCH1, which initiates the production of BH4 and neopterin, has its expression regulated by phenylalanine availability however it is also highly induced by pro-inflammatory cytokines IFN-γ and tumor necrosis factor-α (TNF-α).^43^ During states of inflammation, the production of BH4 is diminished in favor of neopterin and as a result, the breakdown of AAAs is inhibited.^43^ IFN-γ is also implicated in oxidative stress by inducing reactive oxygen species (ROS) that oxidize BH4 thus removing its functionality.^44^ Furthermore, because NOS production is partly dependent on BH4 availability, nitric oxide (NO) levels are also decreased and their ability to suppress IDO-1 activity is impaired.^43^ As a result, tryptophan breakdown in the kynurenine pathway is accelerated and kynurenine is further metabolized to cytotoxic molecules.^43,45^

Tryptophan is an important amino acid of the gut-brain axis. While up to 90% of the kynurenine pathway is metabolized in the liver, a small portion of it, as well as the majority of serotonin, is metabolized in the gastrointestinal tract (GI).^46^ There, besides kynurenine metabolites, tryptophan is also degraded to the neurotransmitters serotonin and melatonin whose dysregulation is responsible for sleep and mood disorders.^47,48^ Additionally, tryptophan is a precursor to the indole and indole-derivative pathways in the GI.^46^ These metabolites also take up various roles in assisting the body’s immune response and modulating inflammation.^49^ Indole and indole-3-aldehyde (I3A) are agonists of the aryl hydrocarbon receptor (AhR) which regulates the expression of interleukin-22 (IL-22) and combats pathogens by promoting mucosal integrity.^50^ Indole also helps maintain the intestinal epithelial barrier and modulates the expression of pro- and anti-inflammatory genes.^50^ The final indole-derivative, indole-3-propionate (IPA), exerts neuroprotective and antioxidant effects.^50^ In pathological states the balance of tryptophan metabolism shifts away from these metabolites and in favor of the kynurenine pathway resulting in a fertile ground for the development of inflammation and its derivative disorders.^15,51,52^

The processes described above take place in the GI and, as a result, the gut microbiome is a relevant factor in the metabolic pathways of tryptophan. A dietary lack of tryptophan has been shown to alter microbiota composition in the GI.^53^ Alterations in the gut microbiome have been associated with diminished gut barrier function in inflammatory diseases.^54^ The microbial species taking part in the metabolism of indole and its derivatives, include *Bacteroides*, *Bacillus,* and *Clostridium*.^51,52^ Microbiome studies in women with PCOS have reported reduced alpha diversity in microbiota and dominance of the *Bacteroides* species along with decreased glucagon-like peptide-1 (GLP-1) and neurotransmitter levels.^55−58^ GLP-1 stimulates the secretion of insulin against obesity and its production is modulated by indole.^51^ If we attempted to apply these findings to PCOS pathophysiology, we could say that abnormal intake of tryptophan calls for the overexpression of IDO through pro-inflammatory signals. This process shifts the metabolic scale away from the indole pathway, which compromises gut immunity, altering the intestinal microbiome and further exacerbating the pre-existing inflammation. This prolonged inflammatory response, along with oxidative stress, gives rise to insulin resistance and leads to the eruption of PCOS.

Alterations in gut microbiota composition as a result of AAA imbalance can have repercussions in other metabolic pathways that are relevant in PCOS pathophysiology, namely, the metabolism of bile acids (BAs). These amphiphilic steroid compounds take on important roles with regards to triglyceride metabolism, glucose absorption but also hormonal signaling relating to androgen synthesis and ovarian development.^59^ AAAs have been shown to stimulate BA synthesis under a high-fat diet by disrupting BA metabolism.^60^ Primary BAs are produced in the liver before entering the intestine and having their metabolism initiated by gut microbiota-derived bile salt hydrolases (BSHs) in order to form secondary BAs.^59^ The species involved in this process include *Bacteroides*, *Bacillus,* and *Clostridium* among others,^59^ which are also implicated in the metabolism of indole as described above. A number of studies have shown the dominance of the *Bacteroides* species in PCOS women alongside perturbations of BA metabolism.^59,61^ One such study conducted on Chinese women from Qi et al., reported that the genus *Bacteroides Vulgatus* was overabundant and negatively correlated with glycodeoxycholic acid (GDCA) and tauroursodeoxycholic acid (TUDCA) in PCOS.^62^ At the same time, a reduction in the levels of these BAs was associated with decreased IL-22 secretion,^62^ providing another potential pathway for the AAA-IL-22 axis other than the activation of the AhR that was mentioned before. Although the *Bacteroides* species takes part in both indole and BA metabolism, it appears that its dominance in the gut microbiome of women with PCOS has adverse effects, possibly because these processes require the action of multiple species in different steps of the metabolic pathways. Therefore, the gut microbiota dysbiosis, as a result of AAA perturbations, can lead to the disruption of other important metabolic pathways such as BAs, and it is a crucial aspect of PCOS that necessitates further exploration.

The majority of studies included in this review showed that AAAs were significantly increased in women with PCOS accompanied by increased insulin resistance. Insulin is widely known for its role in glucose uptake but can also act directly on ovarian cells to increase luteinizing hormone (LH) and consequently androgen synthesis.^1^ Furthermore, it takes part in the hypothalamic-pituitary system by enhancing the release of gonadotropin-releasing hormone (GnRH) and subsequently increasing LH-derived androgens as well as increasing adrenal androgen levels.^1^ In addition, it can elevate adrenal androgens by increasing the sensitivity of the adrenal glands to adrenocorticotropic hormone (ACTH).^1^

The metabolism and physiological function of AAAs are strongly linked to insulin activity and thus to the regulation of glucose homeostasis and the homeostasis of protein metabolism. After nutrient intake, insulin is released from the β-cells of the pancreas as a result of the presence of glucose in the bloodstream. The release of insulin is further enhanced by the presence of amino acids and in particular AAAs.^63^ Tryptophan and phenylalanine, in particular, can bind to the G protein-coupled receptor 142 (GPR142) in pancreatic cells which stimulates the release of insulin as well as the incretin hormones gastric inhibitory polypeptide (GIP) and GLP-1 thus improving glucose and amino acid uptake throughout the body.^64^ After nutrient intake, insulin is released leading to the activation of insulin receptor (IR) and insulin-like growth factor-1 receptor (IGF-1R) in cells.^65^ These actions cause a signaling cascade that results in the activation of the mammalian target of rapamycin (mTOR) complex and subsequently protein synthesis while the amino acid uptake into the muscles is upregulated.^63,65^ At the same time, the postprandial abundance of amino acids triggers a structural change that localizes mTOR to the lysosomal surface and enables its activation.^65^ However, during an insulin-resistant state, the defects in these signaling pathways impair insulin’s function leading to diminished protein synthesis and increased circulation of amino acids.^7^

The 3 studies that mapped the metabolic profile of insulin resistance in the syndrome noted that phenylalanine and tyrosine are the molecules most closely implicated with the disorder.^32,33,37^ This finding is in line with Würtz et al.’s proposition that these 2 amino acids predict insulin resistance.^12^ Tyrosine autophosphorylation plays a key role during insulin’s receptor binding, enabling the phosphorylation of intracellular substrates such as insulin receptor substrate-1 (IRS-1) and a subsequent cascade that instigates insulin signal transduction.^1^ A decrease in the initial step of tyrosine autophosphorylation along with an increase in serine phosphorylation can cause the inhibition of insulin action as a result of postbinding defects.^1,66^ It has been suggested that this mechanism of disorder in the insulin receptors is a unique trait of PCOS not observed in other metabolic abnormalities.^66^ Moreover, a study on mice showed that intracellular phenylalanine excess could block insulin signaling by modifying the β subunit of the insulin receptor in response to amino acid overnutrition.^67^ Similarly, excess tryptophan was also found to modify insulin signaling in mice and in human adipocytes *in vitro*.^68^ Another mechanism that interposes tyrosine’s effectiveness during insulin signaling is its replacement by the isoforms ortho- (o-Tyr) and meta-tyrosine (m-Tyr).^69^ The reason for this is the oxidation of phenylalanine’s aromatic ring by hydroxyl free radicals leading to the formation of these nonphysiological isomers of tyrosine.^69^ As a result, they take up the functioning role of tyrosine residues in IRS-1 however they exhibit worse binding affinity and poor substrate capability leading to insulin resistance.^69^

In the literature, AAAs are often mentioned alongside another group of proteinogenic compounds, the branched-chain amino acids (BCAAs) leucine, isoleucine, and valine, which are also derived strictly from diet like AAAs. BCAAs exhibit important functions in the human body ranging from protein synthesis and glucose metabolism to improving immune response and regulating neurotransmitter synthesis.^70^ Furthermore, their perturbations have been implicated in conditions such as obesity, type 2 diabetes mellitus, insulin resistance but also PCOS.^71,72^ In the context of PCOS pathophysiology, the interplay between the two groups of amino acids presents great interest, because these metabolites share common signaling pathways. First of all, AAAs and BCAAs have a common transporter in the blood–brain barrier, whose course of action is dictated by the concentration of each amino acid.^73^ Tyrosine and tryptophan utilize this transporter in order to commence neurotransmitter synthesis, however, they have to compete for a spot in the “vehicle” with BCAAs thus diminishing AAA uptake and neurotransmitter levels.^73^ Moreover, BCAAs can activate the mTOR pathway and induce serine phosphorylation of IRS-1 and IRS-2 leading to insulin resistance as described previously.^74^ Indeed, many studies have reported that BCAA levels are increased in PCOS alongside AAAs,^27,30,32,33,75^ suggesting a defective degradation process that impacts women with PCOS metabolically but also through reduced neurotransmitter availability. Taking into account the competing nature of these metabolites when it comes to cellular signaling, we can hypothesize that the interactions between the perturbations of AAAs and BCAAs have a significant role in PCOS pathology; thus, their metabolism could be investigated in parallel in future metabolomics studies.

Focusing on the association between AAAs and insulin resistance reveals that sexual dimorphism influences their interaction. A study by Würtz et al. showed that tyrosine’s magnitude of association with insulin resistance was 5 times higher in men than women.^10^ Additionally, when accounting for waist circumference, in women tyrosine showed significance only in the upper tertile in contrast to men where tyrosine was significant in all ranges.^10^ Based on these observations, we can hypothesize that the changes in the hormonal profile of PCOS women also reflect on their metabolic profile. However, it is worth noting that in men insulin resistance and BMI are linked with a decrease in testosterone levels whereas the opposite is true for women.^76^ This could be indicative of other mechanisms affecting the interaction between the metabolic and hormonal phenotypes in women with PCOS.

As mentioned before, insulin resistance is not found in all PCOS cases.^1^ On the contrary, it is hyperandrogenism, ovulatory dysfunction, and, to a lesser degree, polycystic ovarian morphology that are the cornerstones of the syndrome.^2^ A significant amount of evidence supports the involvement of AAA metabolism in the processes underlying the defining traits of PCOS.

One of the mechanisms through which androgen synthesis occurs is the stimulation of GnRH and LH to produce testosterone.^77,78^ Although physiologically these 2 hormones produce both kinds of sex hormones, their immediate effect is to induce a surge in testosterone levels.^79^ Because the activation of GnRH and LH occur in a pulsatile manner, an increase in the rhythm of these pulses also leads to more frequent surges of testosterone.^1,77^ Under normal circumstances, the frequency of GnRH release is decreased by testosterone.^80^ However, this is not true in PCOS, suggesting that the overall regulation of the hormone’s activity is impaired. Serotonin and catecholamines, the metabolites of AAAs, are neurotransmitters that among other functions regulate the activity of GnRH and LH.^81^ Serotonin can both inhibit and stimulate GnRH release by activating either the serotonin 1A (5-HT_1A)_ or serotonin 2A (5-HT_2A)_ receptors in neurons, respectively.^82^ Norepinephrine acts on the α- and β-adrenergic receptors to block and stimulate LH release while epinephrine utilizes the α-adrenergic receptor to stimulate the release of both GnRH and LH.^81^ On the other hand, dopamine has a major suppressing effect on GnRH release by activating the D1 and D2 receptors.^83^ In a model of PCOS rats, the brain levels of the AAA-derived neurotransmitters as well as the expression of 5-HT_1A_, α-adrenergic receptor, and D2 receptor were decreased thus elevating GnRH and LH release and increasing androgen levels.^81^ Hence, it can be deduced that the inability of AAAs to be metabolized into these neurotransmitters has important consequences in hormonal regulation that lead to the emergence of hyperandrogenism.

Regarding ovulatory dysfunction, studies in animals have highlighted the role of serotonin in oocyte development.^84,85^ The secretion of progesterone in ovarian granulosa cells is regulated by serotonin.^85^ Progesterone is a hormone that is important in reproductive health.^86^ The serotonergic signals are carried out by the neurotransmitter’s binding to 5-HT receptors in cumulus cells and oocytes with the help of the membrane serotonin transporter (SERT).^85,87^ This results in the increase of Ca^2+^ and cyclic adenosine monophosphate (cAMP) activity, 2 important mediators in oocyte maturation signaling.^88,89^ In contrast, kynurenine was negatively correlated with progesterone in postpartum women while progesterone has been known to inhibit IDO activity.^90,91^ Moreover, a study by Bódis et al. noted that achieving a successful pregnancy involved a shift toward the serotonin pathway instead of kynurenine.^92^ These findings suggest that the disruption of tryptophan’s metabolic balance has adverse effects on oocyte development resulting in ovulatory perturbations in patients with PCOS.

The significant amount of evidence toward AAA disorders in PCOS compels us to explore any potential genetic links between their metabolic pathways and the syndrome. A comprehensive search of genomewide association studies (GWAS) on PCOS did not identify any loci directly linking aromatic amino acids or their metabolic pathways to the condition. While GWAS has uncovered various genetic factors associated with PCOS, no explicit connection to aromatic amino acid metabolism has been reported to date. On the other hand, various GWAS have reported single nucleotide polymorphisms (SNPs) in the gene encoding insulin receptors,^93−95^ thus providing a potential avenue connecting AAAs and PCOS in a genetic level. However, evidence regarding the significance of these SNPs in PCOS has been conflicting.^93,96,97^ Moreover, we cannot be certain that the pathological mechanism emerging from these mutations is associated with AAA perturbations. Therefore, the genetic association between AAA metabolism and PCOS is a matter that warrants further exploration in the future.

**Figure 3:**
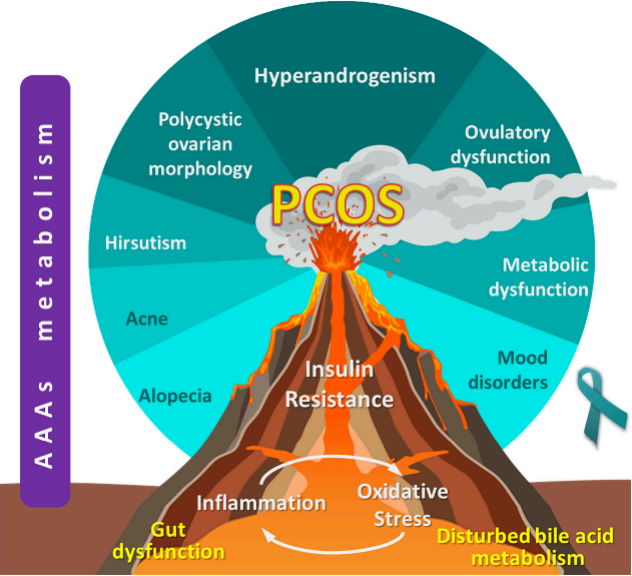
Inflammation and oxidative stress form a feedback loop, inducing the onset of insulin resistance. Insulin resistance is considered a precursor of polycystic ovary syndrome (PCOS). Once PCOS is established, apart from the diagnostic criteria, hyperandrogenism, ovulatory dysfunction, and polycystic ovarian morphology, it may encompass a multitude of symptoms such as hirsutism, acne, alopecia, metabolic dysfunction, and mood disorders. Aromatic amino acid (AAAs) metabolism perturbations have been reported in PCOS but also mark inflammation, oxidative stress, and insulin resistance.

As depicted in Figure 3, it becomes apparent that AAAs hold great importance in the pathologies of PCOS, thus implying their potential to be explored as diagnostic markers in the disease. Their diagnostic strength in metabolic disorders and irritable bowel syndrome is well-documented.^17,98^ Within the scope of our review, 2 studies examined phenylalanine-tyrosine and tryptophan separately and found that they can form diagnostic signatures for PCOS perhaps even as early predictors for the onset of the disease.^33,40^ Because AAAs and other amino acids are greatly associated with obesity and insulin resistance, the question of whether these diagnostic signatures are indicative of PCOS itself and not of a metabolic morbidity of the syndrome is a matter that solicits further exploration. Current literature suggests that AAA metabolism disorders in PCOS are not exclusively associated with BMI and insulin resistance status. A 2020 study conducted on follicular fluid of PCOS patients with normal BMI and no insulin resistance reported increased tyrosine and tryptophan levels and also strong correlations between AAA levels and serum concentration of androgens.^27^ An earlier study from 2014 showed that AAAs were increased in PCOS even when adjusting for BMI however, BCAAs were upregulated in patients with high BMI regardless of PCOS status.^75^ Additionally, in the same study, non-insulin-resistant patients exhibited higher AAA levels compared to non-insulin-resistant controls, further confirming the strong link of these metabolites with PCOS irrespective of metabolic status.^75^ Finally, a study by Zhao et al. showed that alongside amino acid perturbations, the BCAA/AAA ratio was significantly reduced in PCOS and not impacted by obesity or insulin resistance.^30^ Considering these findings, we are led to believe that AAA signatures are important in the pathology of PCOS even when metabolic symptoms are left out of the picture.

Diagnosing PCOS presents many challenges owing to the discord between proposed criteria and subsequently the accepted phenotypes but also the use of suboptimal means for hormonal assessment.^2^ Although PCOS is described as both an endocrine and metabolic disorder, the endocrine aspect often takes precedence, leaving metabolism an afterthought. To that end, the strength of AAAs as diagnostic markers and their ability to detect abnormalities could pave the way for a revision of the modern PCOS guidelines. This becomes all the more important when considering that much of the syndrome’s treatment lies in lifestyle modification and consequently a timely intervention could provide a vastly improved quality of life for the affected population.^2^

Treatment goes along with diagnosis, and exploring AAAs and their metabolic pathways for this purpose is of vital importance. Exenatide is a GLP-1 agonist that has shown good results in the treatment of metabolic disorders and is emerging as a promising treatment for PCOS as well.^42,99,100^ According to Tang et al., exenatide acts by alleviating amino acid disorders with phenylalanine and tyrosine holding a prominent place in its pharmacodynamics.^42^ Another widely used treatment for PCOS is contraceptive pills. Combined oral contraceptives (COCs) are a common intervention for the amelioration of hormonal symptoms in PCOS.^101^ However, concerns have been raised about their safety regarding insulin sensitivity, lipid profile, cardiovascular diseases, and venous thrombotic events (VTEs).^2,101,102^ In our systematic review, one study assessed the effect of COCs on women with PCOS and normal BMI and although there was an improvement in some hormonal and metabolic indices there was also an enrichment in the aromatic amino acid pathways.^41^ Therefore, exploring aromatic amino acid pathways as targets for the treatment of PCOS could lead to novel and more effective therapies in the future.

**Figure 4:**
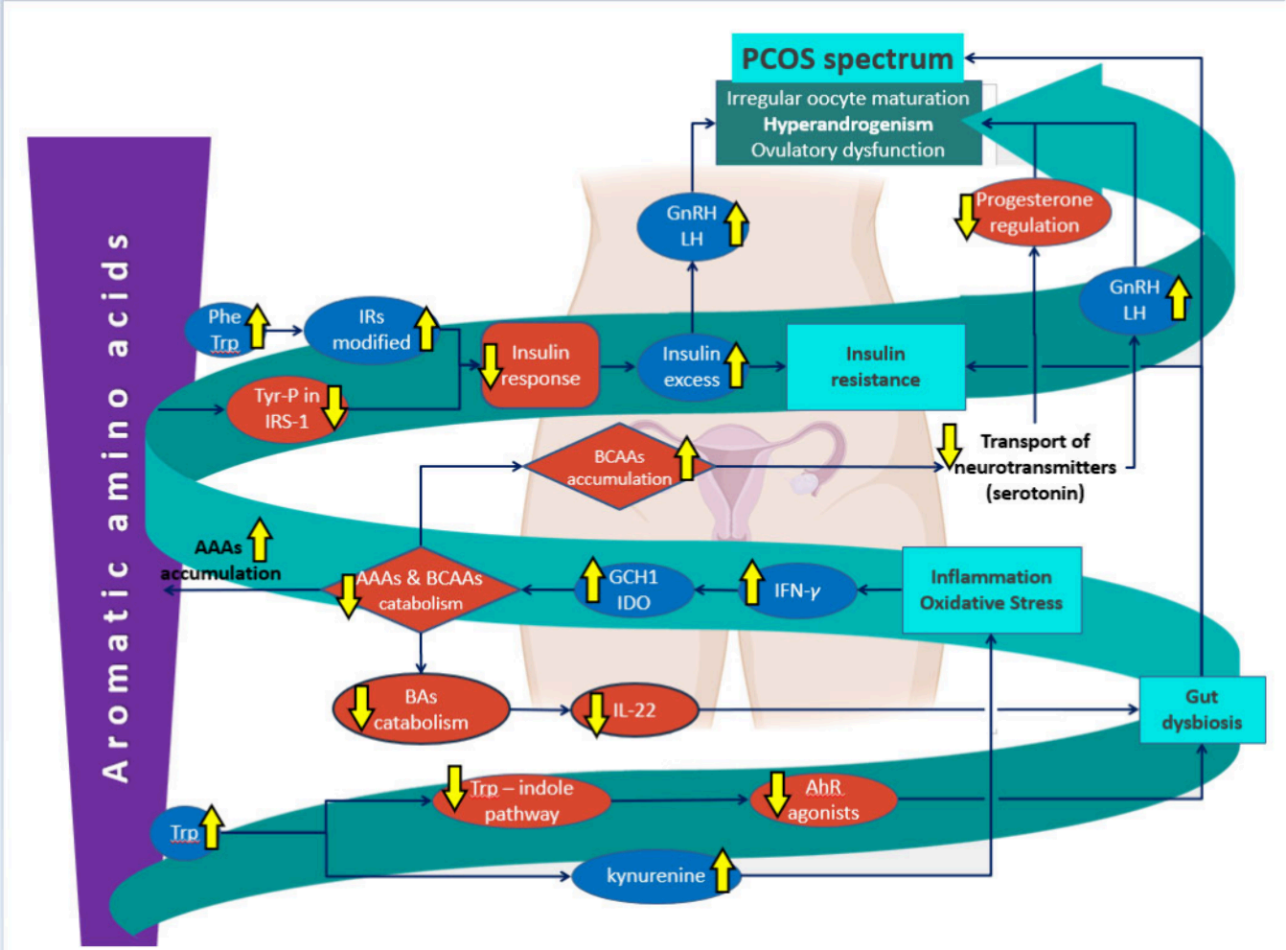
Suggested biochemical pathways of AAAs’ influence on PCOS. Phe: phenylalanine, Trp: tryptophan, IRs: insulin receptors, Tyr-P: tyrosine phosphorylation, IRS-1: insulin receptor substrate-1, GnRH: gonadotropin-releasing hormone, LH: luteinizing hormone, PCOS: polycystic ovary syndrome, AAAs: aromatic amino acids, BCAAs: branched-chain amino acids, GCH1:GTP-cyclohydrolase 1, IDO: indoleamine-2,3-dioxygenase, IFN-γ: interferon-γ, BAs: bile acids, IL-22: interleukin-22, AhR: aryl hydrocarbon receptor.

In summary, as illustrated in Figure 4, multiple papers provide evidence that AAA perturbations are detected in every stage of PCOS pathophysiology, from inflammation and oxidative stress to the development of insulin resistance and through that to the hormonal and ovulatory dysfunctions that mark PCOS. The prodrome step in the ascending spiral of PCOS is inflammation along with oxidative stress. IFN-γ activates the GCH1 and IDO enzymes, disrupting AAA metabolism and limiting their conversion into neurotransmitters. In the case of tryptophan, this translates to a shift of metabolic balance toward the kynurenine pathway. This shift reduces the production of serotonin and other indole derivatives, leading to an imbalance in the tryptophan-indole pathway. This imbalance leads to immune dysregulation and gut dysbiosis, as the reduced availability of aryl hydrocarbon receptor (AhR) agonists impairs mucosal integrity and inflammatory control, further worsening inflammation. This inflammatory loop develops into insulin resistance. AAA abnormalities inhibit insulin signaling by reducing tyrosine’s phosphorylation capability in IRS-1. Additionally, modifications in phenylalanine and tryptophan can impair insulin receptor function, further disrupting insulin signaling. The resulting hyperinsulinemia stimulates GnRH and LH activity, leading to increased androgen synthesis, irregular follicle maturation, and ovulatory dysfunction. Ιnsulin resistance is not present in all cases. Apart from the pathway of inflammation-insulin resistance, AAA perturbations also directly impact PCOS symptoms. Hyperandrogenism, ovulatory dysfunction, and polycystic ovarian morphology remain the defining features. Reduced neurotransmitter availability, particularly serotonin, results in increased GnRH and LH release, promoting hyperandrogenism. Moreover, diminished serotonin levels impair the hormone’s ability to regulate progesterone secretion, leading to irregular oocyte development.

The fact that the present review focuses on PCOS mechanisms through aromatic AAAs using recent studies from the past five years, which, while providing an up-to-date perspective, may narrow the scope, thus presenting a limitation of the study. Moreover, it emphasizes blood samples (serum and plasma) and excludes other matrices such as follicular fluid or tissue biopsies, which could enrich understanding. Many studies are cross-sectional, which limits causal insights; future longitudinal research would be beneficial. Additionally, except for Escobar-Morreale and Martinez-Garcia et al.,^34,35^ most studies did not consider nutritional status, which could further clarify the link between diet and PCOS.

## 5 Conclusion

Current evidence supports that the levels of AAAs are increased in women with PCOS as a result of dysregulations in their metabolic pathways. AAAs and their pathways are linked with various facets of the syndrome, with the main one being the development and perpetuation of insulin resistance along with hormonal imbalances but also comorbidities such as chronic inflammation, obesity, and dysregulation of the gut-brain axis. Moreover, they show promising results as diagnostic biomarkers and also as therapeutic targets of novel drug treatments such as exenatide. Furthermore, the perturbations of AAAs are implicated in every stage of the disease pathophysiology, from the underlying onset of inflammation and oxidative stress to the development of insulin resistance and the establishment of the syndrome’s defining traits. Based on our current knowledge, we can hypothesize that AAA perturbations are strongly correlated with PCOS though it remains to be investigated whether they provide the driving force or if they are just another cog in a dysfunctional machine. For all the above, AAAs could prove a promising target for future clinical studies to further determine their value as a distinct entity in understanding the vast spectrum of polycystic ovary syndrome comorbidity, symptoms, and etiology.
